# Identifying Cancer Specific Driver Modules Using a Network-Based Method

**DOI:** 10.3390/molecules23051114

**Published:** 2018-05-08

**Authors:** Feng Li, Lin Gao, Peizhuo Wang, Yuxuan Hu

**Affiliations:** School of Computer Science and Technology, Xidian University, Xi’an 710071, China; lifeng_10_28@163.com (F.L.); wangpeizhuo_37@163.com (P.W.); yuxuan_hu_xd@163.com (Y.H.)

**Keywords:** cancer, network, driver module, specific driver module, driver gene

## Abstract

Detecting driver modules is a key challenge for understanding the mechanisms of carcinogenesis at the pathway level. Identifying cancer specific driver modules is helpful for interpreting the different principles of different cancer types. However, most methods are proposed to identify driver modules in one cancer, but few methods are introduced to detect cancer specific driver modules. We propose a network-based method to detect cancer specific driver modules (CSDM) in a certain cancer type to other cancer types. We construct the specific network of a cancer by combining specific coverage and mutual exclusivity in all cancer types, to catch the specificity of the cancer at the pathway level. To illustrate the performance of the method, we apply CSDM on 12 TCGA cancer types. When we compare CSDM with SpeMDP and HotNet2 with regard to specific coverage and the enrichment of GO terms and KEGG pathways, CSDM is more accurate. We find that the specific driver modules of two different cancers have little overlap, which indicates that the driver modules detected by CSDM are specific. Finally, we also analyze three specific driver modules of BRCA, BLCA, and LAML intersecting with well-known pathways. The source code of CSDM is freely accessible at https://github.com/fengli28/CSDM.git.

## 1. Introduction

Cancer is considered as a complex disease driven by genome alterations that include gene mutations, copy number alterations, and so on [1,2]. A key challenge is to detect driver mutations, which contribute to the development of cancer, from passenger mutations, which are functionally neutral [3]. There are many frequency-based methods that have been proposed to detect significantly mutated genes or predict driver genes by estimating the background mutation rate, such as MuSiC [4], MutSigCV [5,6], ActiveDriver [7], OncodriveClust [8], OncodriveFM [9], OncodriveFML [10], TUSON [11], 20/20+ [12], and CompositeDriver [13]. Tokheim et al. [12] propose a machine learning-based method for driver gene prediction, and also establish an evaluation framework to compare the performance of eight prediction methods. They show that driver genes predicted by each of the eight methods vary widely, and most current methods do not fully consider the mutational heterogeneity [12]. Some other methods prioritize driver genes based on mutation data and functional networks [14,15] or matrix factorization framework [16,17,18,19,20]. However, these methods also do not consider the complicated mutational heterogeneity among patients [3,21,22,23]. Since the genes with driver mutations always work together in cellular signaling and regulatory pathways [21,24], detecting driver pathways, driver modules or driver gene sets, with genes possessing driver mutations, can consider this complicated mutational heterogeneity and provide an understanding of carcinogenesis at the pathway level.

There are mainly two classes of approaches which are proposed to detect driver pathways, driver modules, or driver gene sets: de novo identification approaches and prior knowledge-based methods [25,26]. De novo methods identify combinatorial patterns of cancer mutations without any prior knowledge but utilize two properties of a driver gene set: high coverage and high mutual exclusivity [27,28,29,30,31,32,33,34]. High coverage means that a driver gene set should cover a large number of samples. High mutual exclusivity means that a gene with a driver mutation involved in a pathway is enough to disturb this pathway [22,23]. For example, Dendrix [27] detects combinations of genes that have high coverage and high mutual exclusivity by solving a maximum coverage exclusive submatrix problem. MDPFinder [28], Multi-Dendrix [29], ComMDP, and SpeMDP [34] use integer linear program to solve the maximum coverage exclusive submatrix problem for identifying mutually exclusive sets of genes. Prior knowledge-based methods use the known interaction networks to identify significantly mutated subnetworks or driver modules with mutually exclusive mutated genes [35,36,37,38]. For example, MEMo [36] detects network cliques of mutated genes with mutually exclusive patterns across multiple patients. MEMCover [37] combines tissue type exclusivity with interaction data to detect mutually exclusive groups of mutated genes in the same or across different tissues. HotNet2 [38] utilizes insulated network diffusion to identify significantly mutated subnetworks, which captures the directionality of interactions.

However, different cancer types may have different principles at the pathway level, which is critical for personalized therapy and precision medicine in cancer treatment. Furthermore, several studies suggest there are differences between different cancer types. Bailey et al. [13] give a comprehensive analysis of oncogenic driver genes and mutations across 33 cancer types, and identify 299 cancer driver genes. They find that 142 genes are associated with a single cancer type, which is likely to be the specific genes, while 87 genes are associated with two or more cancer types. For example, TP53 is a tumor suppressor gene, and is associated with 27 cancer types, which is likely to be a common gene. Thus, different cancer types may have common driver genes and specific driver genes, while different genes may play different roles in different cancer types. Sanchez-Vega et al. [39] present an integrated analysis of genetic alterations in ten signaling pathways across 33 cancer types, which denotes that different cancer types have similarities and differences in frequencies of alteration of individual pathways. Thus, different cancer types may have different principles at the pathway level, which is critical for personalized therapy and precision medicine in cancer treatment [40,41]. Therefore, detecting the cancer specific driver modules, including specific genes, is important to understand the different mechanisms of different cancers at the pathway level. However, few methods are introduced to detect specific driver modules for a certain cancer to other cancer types. The specific driver modules for a certain cancer type to other cancer types are different from the driver modules detected in a single cancer type. The driver modules detected in a single cancer type always contain both specific and common parts. There are several methods for identifying specific modules or patterns in multiple cancers [42,43,44,45], but few methods are proposed to detect specific driver modules for a certain cancer type to other cancer types. SpeMDP is an optimization model to discover specific driver gene sets, de novo, of one certain or multiple cancer types, to other cancers with a fixed module size [34]. 

In this work, we propose a network-based method to detect cancer specific driver modules (CSDM), which can catch the specificity of a certain cancer type to other cancer types at the pathway level. A cancer specific driver module must have high coverage and high exclusivity in a certain cancer, and a higher percentage of samples in this cancer than other cancer types. We first construct the specific network for a certain cancer type by integrating specific coverage and mutual exclusivity in all cancer types. Then, we use a greedy algorithm to detect all of the specific driver modules in the specific network. We apply CSDM on 12 TCGA cancer types, and compare it with HotNet2 and SpeMDP on specific coverage and F-measure of GO and KEGG pathway enrichment. We also investigate the overlaps between the specific driver modules of every two different cancer types. Then, we also analyze three different cancer specific driver modules of three cancers, BRCA, BLCA, and LAML.

## 2. Results

In this section, we first compare CSDM with other two methods, HotNet2 and SpeMDP, based on 12 TCGA cancer types. These three methods are compared on their specific coverage and F-measure of pathway enrichment. Then, we analyze three specific driver modules of BLCA, BRCA, and LAML detected by our method based on 12 TCGA cancer types, respectively.

### 2.1. Comparison Study

We first compare CSDM with HotNet2 [38] and SpeMDP [34], based on 12 TCGA cancer types. HotNet2 is a famous method to identify significantly mutated subnetworks, and SpeMDP is an algorithm for identifying a certain or multiple cancer specific driver gene sets. CSDM detects the specific driver modules for a certain cancer type to other cancer types. We compare the results of these three methods with regard to their specific coverage and F-measure of pathway enrichment. First, we use the specific coverage to evaluate whether the driver modules are specific in a particular cancer. Then, we utilize the F-measure of Gene Ontology (GO) terms [46] and Kyoto Encyclopedia of Genes and Genomes (KEGG) [47] pathway enrichment, which are downloaded from Molecular Signatures Databases (MSigDB) [48,49], to evaluate the performance of methods.

#### 2.1.1. Comparison of Specific Coverage

**Figure 1:**
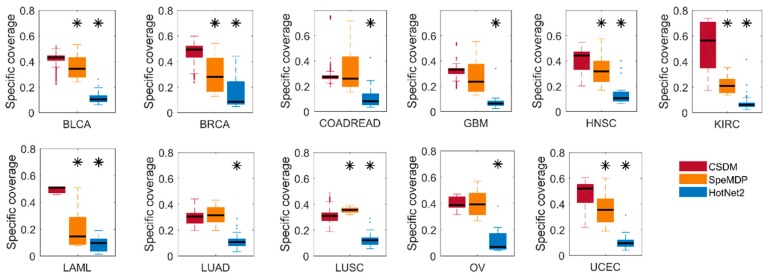
Comparison of CSDM (red), SpeMDP (yellow), and HotNet2 (blue) and their specific coverage in 11 different cancer types. The stars above the blue and red bar represent the differences between CSDM and SpeMDP and HotNet2 in specific coverage, which are computed using Fisher’s exact test (* *p* < 0.05). The confidence intervals for the boxplots correspond to q1 − 1.5 × (q3 − q1) and q3 + 1.5 × (q3 − q1), where q1 and q3 are the 25th and 75th percentiles, respectively.

We compare CSDM, SpeMDP, and HotNet2 in their specific coverage, in Figure 1. Specific coverage is used to measure the specificity of the modules for a cancer type to other cancer types. Obviously, CSDM has significantly higher specific coverage than SpeMDP in seven cancer types, while it has similar specific coverage in other four cancers. Overall, CSDM has significantly higher specific coverage than HotNet2 in all cancer types. HotNet2 has the minimum specific coverage when compared with SpeMDP and CSDM in all cancer types. The main reason is that HotNet2 is a method for identifying significantly mutated subnetworks in a single cancer type, which may mix some specific and common modules. Therefore, it also explains the differences between the specific driver modules of a certain cancer to other cancer types, and the driver modules detected in a single cancer. In general, CSDM can detect cancer specific driver modules with higher specificity than HotNet2 and SpeMDP.

#### 2.1.2. Comparison on Pathway Enrichment

**Figure 2:**
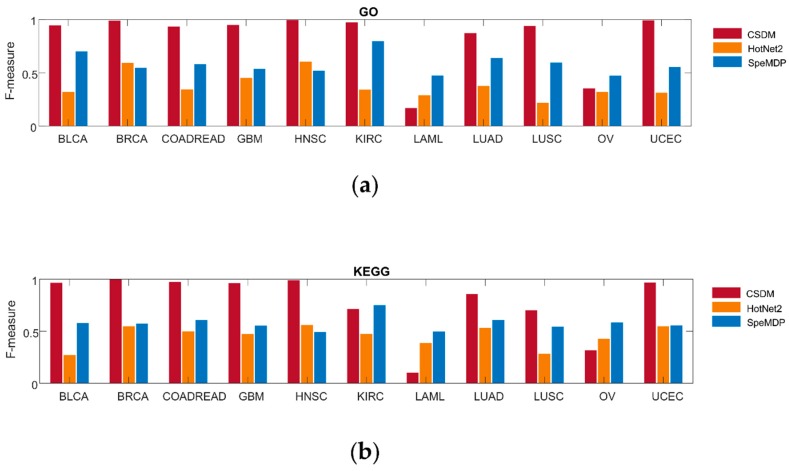
Comparison of CSDM (red), SpeMDP (yellow), and HotNet2 (blue) in GO (**a**) and KEGG (**b**) pathway enrichment in 11 different cancer types. The F-measure is used to represent the accuracy of the driver modules detected by each method.

We also compare CSDM, SpeMDP, and HotNet2 on enrichment of GO terms and KEGG pathways in Figure 2. For the pathway enrichment, we use the Gene Set Enrichment Analysis (GSEA) [48] to obtain the significance *p*-value of a driver module based on the well-known pathway. If *p*-value < 0.05, this driver module is considered to be significant based on pathway enrichment. We use F-measure to represent the accuracy of the driver modules. F-measure is the harmonic mean of precision and recall. The higher the F-measure, the more the driver modules can be enriched to the known pathways. CSDM has a higher F-measure than SpeMDP and HotNet2 in nine cancer types when comparing enrichment of GO terms, while it has the higher F-measure than SpeMDP and HotNet2 in eight cancer types, when comparing enrichment of KEGG pathways. In general, CSDM has higher accuracy than HotNet2 and SpeMDP, based on pathway enrichment.

### 2.2. Overlaps between Different Cancer Types

We analyze the overlaps between the specific driver modules detected by CSDM in 12 different cancer types. In this work, we consider that two specific driver modules from two different cancers have overlaps if these two specific driver modules have at least one overlapping gene. Then, we use the Jaccard index to count the percentages of the combination of driver modules with overlaps from all possible combinations of two cancer types. Obviously, the driver modules of each combination of two different cancers have very little overlap (Supplemental Table S1), which indicates that the driver modules detected by CSDM are specific for a certain cancer type. We also use the Jaccard index to count the percentages of overlapped genes involved in driver modules between each combination of two cancer types. Obviously, the percentages of overlapped genes between different cancer types are always small (Supplemental Table S2), which indicates that the genes involved in driver modules detected by CSDM often present in a certain cancer type.

### 2.3. Specific Driver Modules in BRCA, BLCA, and LAML

We analyze three cancer specific driver modules detected by CSDM in BRCA, BLCA, and LAML among 12 TCGA cancer types, respectively. The reasons for choosing these three cancers are that BRCA has the maximum number of samples, BLCA has the minimum number of samples, and LAML is the only liquid cancer in all 12 cancer types.

#### 2.3.1. Specific Driver Modules in BRCA

**Figure 3:**
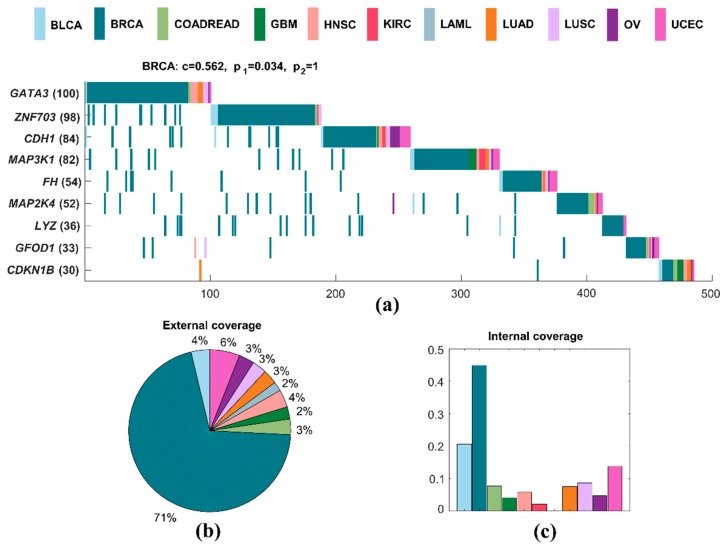
A specific driver module in BRCA. Different colors represent different cancer types. (**a**) Mutation matrix for the cancer specific driver module of BRCA. The genes on the left are the members in the specific driver module. The number behind the gene is the number of samples in which this gene is mutated. (**b**) The external coverage of the specific driver module in (**a**) for each cancer type. (**c**) The internal coverage of the specific driver module in (**a**) for each cancer type.

A specific driver module for BRCA contains nine mutated genes, including *GATA3*, *ZNF703*, *CDH1*, *MAP3K1*, *FH*, *MAP2K4*, *LYZ*, *GFOD1*, and *CDKN1B* (Figure 3a). We use the following measures to analyze the specific driver module. Specific coverage of a module measures the specificity of this module for a particular cancer to other cancer types. The internal coverage of a module is the percentage of mutated samples of this module for a cancer. The external coverage of a module is the fraction of mutated samples of this module for a cancer to all mutated samples of this module for all cancers. The significance of mutual exclusivity is denoted by an empirical *p*-value, which is derived from a random permutation test. It is the fraction of random samples with mutations larger than the real number of samples with mutations in a driver module. The specific coverage of this specific driver module for BRCA is 0.562, while the significance of mutual exclusivity of BRCA is 0.034, and that of all cancers is one. The external coverage of this specific driver module in BRCA is maximum in all cancers, which is 0.705, and means that about 71% of all mutated samples are from BRCA (Figure 3b). The internal coverage of this specific driver module also has the largest value in all cancers, which is 0.448, and denotes that the mutated samples of BRCA accounted for 45% of the total sample size of BRCA (Figure 3c). Obviously, it is a specific driver module for BRCA to other cancer types.

At the same time, this specific driver module intersects with some known pathways. For example, *CDH1*, *MAP3K1*, and *MAP2K4* are members of RAC1 signaling pathway and CDC42 signaling events [50]. *GATA3*, *LYZ*, and *CDKN1B* are members of C-MYB transcription factor network [50]. *CDH1*, *MAP3K1*, *MAP2K4*, and *CDKN1B* are all involved in immune system [51]. *CDH1*, *FH*, and *CDKN1B* are all involved in pathways in cancer [52]. *GATA3*, *ZNF703*, and *MAP3K1* are in the group 1 genes associated with acquired endocrine therapy resistance in breast tumors expressing *ESR1* and *ERBB2* [53]. About a third of the samples in which *GFOD1* is mutated are samples of BRCA. There is no clear evidence that this gene is linked to breast cancer. However, *GFOD1* is significantly upregulated in clear cell renal cell carcinoma tissues, but gradually decreased during cancer progression [54]. 

In order to further study the functional enrichment of the driver module, we use a topology-based pathway analysis method, Mirna enrIched pathway Impact anaLysis (MITHrIL) [55], to analyze this driver module. MITHrIL is an extension of Draghici et al. [56] and Tarca et al. [57], which takes, as input, the expression values of genes and/or microRNAs. The method returns a list of pathways sorted according to their degree of deregulation, together with the corresponding statistical significance (*p*-values). It is capable of clearly improving the reliability of pathway-based analysis of phenotypes. The impact factor reflects the importance of the changes observed in a pathway. The greater the value, the most significant are the changes. Then, we apply MITHrIL on the driver module (*GATA3*, *ZNF703*, *CDH1*, *MAP3K1*, *FH*, *MAP2K4*, *LYZ*, *GFOD1*, and *CDKN1B*) of BRCA using standard KEGG pathways and obtain nine significant pathways (Table 1). Obviously, these nine enriched pathways for BRCA driver module have high impact factor, and are statistically significant (*p*-value < 0.01). 

We also compare CSDM with SPECifIC [58] to study the difference between the cancer specific driver modules using mutation data and the specific subpathways using expression data. All driver genes involved in driver modules detected by CSDM in BRCA are enriched in 107 pathways, with a statistically significant *p*-value < 0.01, while the results obtained by employing SPECifIC in BRCA are enriched in 73 pathways using KEGG pathway terms with the statistically significant *p*-value < 0.01. There are 39 common pathways which are enriched by both two methods. That is to say, these two methodologies provide the same insight in some respects, and can also complement each other in some other respects.

#### 2.3.2. Specific Driver Modules in BLCA

**Figure 4:**
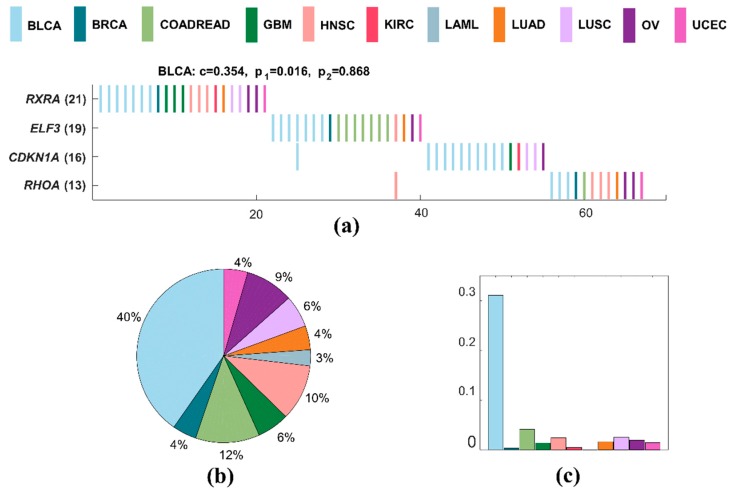
A specific driver module in BLCA. Different colors represent different cancer types. (**a**) Mutation matrix for the cancer specific driver module of BLCA. The genes on the left are the members in the specific driver module. The number behind the gene is the number of samples in which this gene is mutated. (**b**) The external coverage of the specific driver module in (**a**) for each cancer type. (**c**) The internal coverage of the specific driver module in (**a**) for each cancer type.

BLCA has the minimum number of samples in all cancers. A specific driver module for BLCA contains four mutated genes, including *RXRA*, *ELF3*, *CDKN1A*, and *RHOA* (Figure 4a). The specific coverage of this module is 0.562, while the significance of mutual exclusivity of BLCA is 0.034, and that of all cancers is one. This specific driver module has the largest external coverage and internal coverage, with 0.403 (Figure 4b) and 0.310 (Figure 4c) of BLCA, respectively. It means that this module is a specific driver module for BLCA. *RXRA*, *ELF3*, *CDKN1A*, and *RHOA* are all significantly mutated genes related to BLCA [59]. *RXRA*, *CDKN1A*, and *RHOA* are the members of pathways in cancer [52]. *ELF3* and *CDKN1A* are genes downregulated in HeLa cells after knockdown of MED1 by RNAi [60], and they are both regulated by hypoxia [61].

We also apply MITHrIL on the driver module (*RXRA*, *ELF3*, *CDKN1A*, and *RHOA*) of BLCA using standard KEGG pathways and obtain ten significant pathways (Table 2). Obviously, these ten enriched pathways for BLCA driver module have high impact factor and are statistically significant (*p* < 0.01). 

#### 2.3.3. Specific Driver Modules in LAML

**Figure 5:**
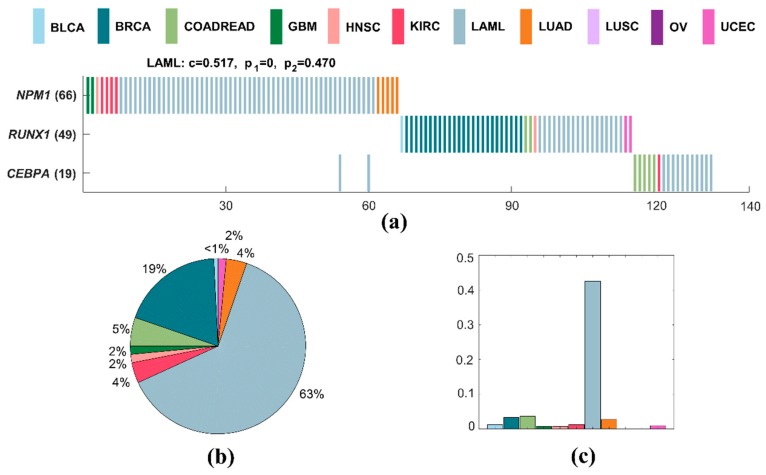
A specific driver module in LAML. Different colors represent different cancer types. (**a**) Mutation matrix for the cancer specific driver module of LAML. The genes on the left are the members in the specific driver module. The number behind the gene is the number of samples in which this gene is mutated. (**b**) The external coverage of the specific driver module in (**a**) for each cancer type. (**c**) The internal coverage of the specific driver module in (**a**) for each cancer type.

A specific driver module for LAML contains three mutated genes, including *NPM1*, *RUNX1*, and *CEBPA* (Figure 5a). The specific coverage of this module is 0.517, while the significance of mutual exclusivity of LAML is 0.016, and that of all cancers is one. This specific driver module has the largest external coverage and internal coverage, with 0.629 (Figure 5b) and 0.427 (Figure 5c) in LAML, respectively. Obviously, it is a specific driver module for LAML compared to other cancer types. The mutations of *NPM1*, *RUNX1*, and *CEBPA* are proven to be associated with LAML [62,63]. *NPM1* is a nucleolar phosphoprotein which plays an essential role in transcription, cell apoptosis, cell proliferation, and the regulation of the p53 pathway [63,64,65]. *RUNX1* mutations predict for resistance to chemotherapy, and they are significantly associated with distinct biological and clinical features [66]. Patients with a biallelic mutation in *CEBPA* are defined as a clinicopathologic entity that is associated with a favorable prognosis [67]. *NPM1*, *RUNX1*, and *CEBPA* are all genes from common genomic gains observed in a meta-analysis of copy number alterations across a panel of different cancer cell lines and tumor samples [68]. *RUNX1* and *CEBPA* are both target genes for *STAT3* in CSF3 signaling during myeloblast differentiation to neutrophils [69].

## 3. Discussion

We present a network-based method (CSDM) to detect specific driver modules of a certain cancer to other cancer types. This method can extract specific parts of a cancer at pathway level. When we apply CSDM on 12 TCGA cancer datasets, CSDM detects specific driver modules successfully. CSDM has higher accuracy than SpeMDP and HotNet2 when comparing specific coverage and GO and KEGG pathway enrichment. Moreover, there are few overlaps between specific driver modules when comparing two different cancers, which indicates that CSDM can get specific driver modules for each cancer type. Lastly, three specific driver modules detected by CSDM in BRCA, BLCA, and LAML intersect with well-known pathways, also verifying the validity of CSDM. When we apply MITHrIL on the specific driver modules of BRCA and BLCA, the enriched pathways have a high impact factor and are statistically significant (*p*-value < 0.01). Moreover, CSDM can be also applied on subtype specific driver modules in one cancer type, in theory. Since the sample size distribution of subtypes for an individual cancer is uneven, and the mutation data itself is very sparse, it is not suitable for CSDM to detect the specific driver modules of subtype only with mutation data. There are mainly two opportunities to improve CSDM in future work. On one hand, other multi-omics data, such as gene expression and DNA methylation, might be integrated into this framework to extract more specific information from different aspects. On the other hand, prior knowledge about the pathways, such as well-known pathways and protein interaction networks, can be used to improve this method and obtain more significantly specific driver modules.

## 4. Datasets and Methods 

### 4.1. Datasets

We use pan-cancer genomic aberration data from 12 cancer types to assess our method for applications, downloaded from The Cancer Genome Atlas (TCGA) (http://cancergenome.nih.gov/), which are also used by HotNet2 [38]. The 12 cancer types are bladder carcinoma (BLCA), breast carcinoma (BRCA), colon adenocarcinoma (COAD), glioblastoma multiformae (GBM), head and neck squamous carcinoma (HNSC), kidney renal clear-cell carcinoma (KIRC), lymphoblastic acute myeloid leukemia (LAML), lung adenocarcinoma (LUAD), lung squamous carcinoma (LUSC), ovarian carcinoma (OV), rectal adenocarcinoma (READ), and uterine cervical and endometrial carcinoma (UCEC). Here, colon adenocarcinoma and rectal adenocarcinoma are combined into one type, denoted as COADREAD. We utilize the same data processing methods introduced by HotNet2 [38].

To represent the mutation data conveniently, we transform the mutation data into a binary mutation matrix. In this paper, we consider K(K≥2) cancer types, and let $S^{\left(k\right)}$ represent the set of samples in the kth caner type with $m_{k}$ elements. Let G represent the set of genes in each caner type with n elements. We use $A^{\left(k\right)}=(a_{ij}^{\left(k\right)})$ to represent the mutation matrix of the kth cancer type. The entry $a_{ij}^{\left(k\right)}=1$ represents that gene $g_{j}$ is mutated in sample $s_{i}^{\left(k\right)}$ and $a_{ij}^{\left(k\right)}=0$ otherwise. There are $m_{k}$ rows (samples) and n columns (mutated genes) in $A^{\left(k\right)}$, where k=1,2,⋯,K. Let $\Gamma^{\left(k\right)}(g_{j})=\{i:a_{ij}^{\left(k\right)}=1\}$ denote the set of samples in which the gene $g_{j}$ is mutated in the kth cancer type [27]. Meanwhile, let a binary matrix *A* = (*a_ij_*) with m rows (samples) and n columns (mutated genes) to represent the mutation matrix for all cancer types, where $m=m_{1}+m_{2}+\cdots +m_{K}$. Let $\Gamma (g_{j})=\{i:a_{ij}=1\}$ denote the set of samples in which the gene $g_{j}$ is mutated in all cancer type [27].

To assess the functional significance of the driver module, we download the gene sets of Gene Ontology (GO) biological process and [46] and Kyoto Encyclopedia of Genes and Genomes (KEGG) [47] from Molecular Signatures Databases (MSigDB) [48,49]. We utilize Gene Set Enrichment Analysis (GSEA) [48] to determine whether a driver module shows statistical significance with *p*-value < 0.05. Then, we utilize the F-measure to evaluate the performance of each method. 

### 4.2. Methods

**Figure 6:**
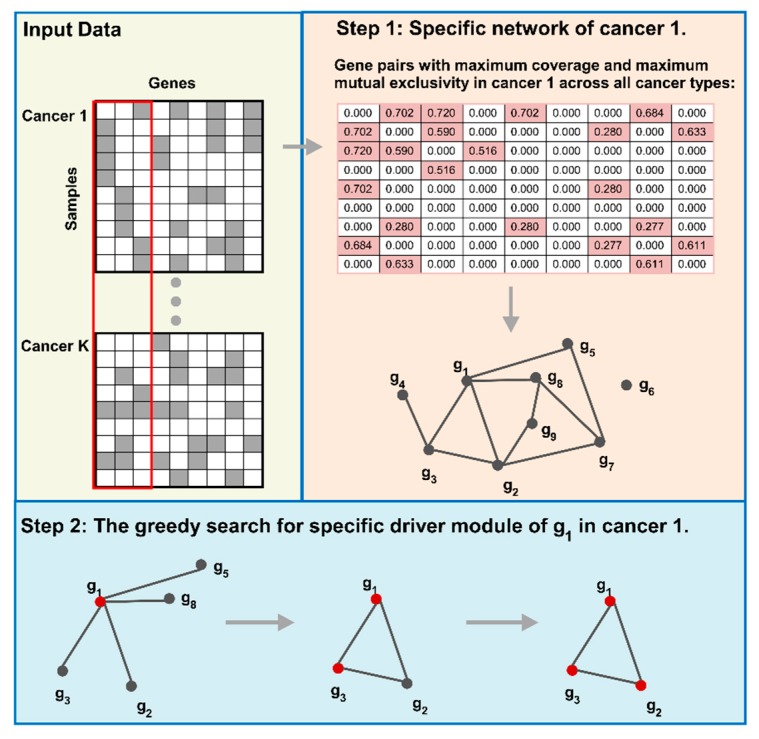
Overview of CSDM. Firstly, we use the binary matrix to represent the mutation data. Then, we construct the specific network for a certain cancer type to other cancer types. At last, we use a greedy search to detect cancer specific driver modules for a certain cancer type.

We first construct the specific network for a certain cancer by integrating specific coverage and mutual exclusivity. Then, we use a greedy search to detect the cancer specific driver modules. The overview of our method is shown in Figure 6. Finally, we utilize the specific coverage and the significance of mutual exclusivity to evaluate the cancer specific driver modules.

#### 4.2.1. Cancer Specific Network Construction

The specific coverage for each gene pair in a certain cancer type is proposed to catch the specificity of the cancer specific driver module. Then, the mutual exclusivity is used to quantify the exclusivity of each gene pair in the same driver module. Finally, a cancer specific network is constructed by combining the specific coverage and mutual exclusivity.

***Specific coverage.*** Given a pair of genes $g_{i},\;g_{j}\in G$, we first define the internal coverage and external coverage of $\left(g_{i},g_{j}\right)$, respectively. The internal coverage of $\left(g_{i},g_{j}\right)$ in cancer k based on K cancer types measures the coverage in cancer k, which is the percentage of samples with at least one mutation in $g_{i}$ or $g_{j}$ in cancer k, defined as follows:

(1) $$c\_in_{ij}^{\left(k\right)}=\frac{\left|\Gamma^{\left(k\right)}(g_{i})\cup \Gamma^{\left(k\right)}(g_{j})\right|}{m_{k}}$$

The external coverage of $\left(g_{i},g_{j}\right)$ in cancer k based on K cancer types measures the relationship between cancer k and all K cancer types. It is the fraction of samples with at least one mutation in gene $g_{i}$ or $g_{j}$ in cancer k, based on all samples with mutations in $g_{i}$ or *g_j_* in K cancer types, defined as follows:

(2) $$c\_ex_{ij}^{\left(k\right)}=\frac{\left|\Gamma^{\left(k\right)}(g_{i})\cup \Gamma^{\left(k\right)}(g_{j})\right|}{\left|\Gamma (g_{i})\cup \Gamma (g_{j})\right|}.$$

The specific coverage of $\left(g_{i},g_{j}\right)$ in cancer k to other K−1 cancer types is the geometric mean of internal coverage and external coverage, and is denoted by $c_{ij}^{\left(k\right)}$, defined as follows:

(3) $$c_{ij}^{\left(k\right)}=\sqrt{c\_in_{ij}^{\left(k\right)}\times c\_ex_{ij}^{\left(k\right)}}.$$

The larger the specific coverage, the more specific the gene pair is to a particular cancer. Then, the specific coverage matrix $C^{\left(k\right)}=(c_{ij}^{\left(k\right)})$ for cancer k is constructed. We normalize it by min-max normalization.

***Mutual exclusivity.*** We utilize a mutual exclusivity index [33] based on an uncertainty coefficient [70] proposed by our another work to quantify mutual exclusivity between each gene pair for each cancer type. This mutual exclusivity index measures the level of mutual exclusivity between two genes, and tends to select gene pairs without a dominating gene, which has a high coverage dominating the total coverage of gene pair. Given a pair of genes $g_{i},\;g_{j}\in G$, the mutual exclusivity of $\left(g_{i},g_{j}\right)$ in cancer k is denoted by $e_{ij}^{\left(k\right)}$, defined as follows [33]:

(4) $$e_{ij}^{\left(k\right)}=\left\{ \begin{array}{l} \frac{u_{ij}^{\left(k\right)}+u_{ij}^{\left(k\right)}}{2},\;s(\alpha_{i}^{\left(k\right)},\alpha_{j}^{\left(k\right)})\leq \min(s({\bar{\alpha }}_{i}^{\left(k\right)},\alpha_{j}^{\left(k\right)}),s(\alpha_{i}^{\left(k\right)},{\bar{\alpha }}_{j}^{\left(k\right)})),\\ 0,\;otherwise. \end{array}\right.$$

and $u_{ij}^{\left(k\right)}$ is the uncertainty coefficient [70] of $\left(g_{i},g_{j}\right)$ in cancer k, and defined as Equation (5), where H is the entropy.

(5) $$u_{ij}^{\left(k\right)}=\frac{H(\alpha_{i}^{\left(k\right)})+H(\alpha_{j}^{\left(k\right)})-H(\alpha_{i}^{\left(k\right)},\alpha_{j}^{\left(k\right)})}{H(\alpha_{j}^{\left(k\right)})}$$

$s(\alpha_{i}^{\left(k\right)},\alpha_{j}^{\left(k\right)})$ is the support degree of $\left(g_{i},g_{j}\right)$ in cancer k and defined as Equation (6).

(6) $$s(\alpha_{i}^{\left(k\right)},\alpha_{j}^{\left(k\right)})=\frac{\left|\alpha_{i}^{\left(k\right)}\cap \alpha_{j}^{\left(k\right)}\right|}{\left|\alpha_{i}^{\left(k\right)}\cup \alpha_{j}^{\left(k\right)}\right|}$$

$\alpha_{i}^{\left(k\right)}=(a_{1i}^{\left(k\right)},a_{2i}^{\left(k\right)},\cdots ,a_{m_{k},i}^{\left(k\right)})$ is the profile of gene $g_{i}$ in cancer k, which is the ith column in $A^{\left(k\right)}$, And ${\bar{\alpha }}_{i}^{\left(k\right)}$ is the complementary profile of $\alpha_{i}^{\left(k\right)}$ in cancer k.

Then, the mutual exclusivity matrix $E^{\left(k\right)}=(e_{ij}^{\left(k\right)})$ is constructed. We also normalize *E*^(*k*)^ using min-max normalization.

***Network construction.*** We construct the specific network of cancer k by combining the specific coverage and mutual exclusivity for each gene pair to other cancer types. 

First, we select the gene pairs that have largest specific coverage and mutual exclusivity simultaneously in cancer k to other K−1 cancer types. These gene pairs are more likely to be the specific gene pairs of cancer k. We use the harmonic mean of the specific coverage and mutual exclusivity to construct the specific network of cancer k, and the weight for each gene pair is calculated by Equation (7).

(7) $$w_{ij}^{\left(k\right)}=\left\{ \begin{array}{l} \frac{2}{1/c_{ij}^{\left(k\right)}+1/e_{ij}^{\left(k\right)}},\;if\;c_{ij}^{\left(k\right)}\geq \underset{1\leq q\leq K}{\max}\{c_{ij}^{\left(q\right)}\},\;m_{ij}^{\left(k\right)}\geq \underset{1\leq q\leq K}{\max}\{e_{ij}^{\left(q\right)}\},\\ 0,\;otherwise. \end{array}\right.$$

To get more precise specific networks, we do z-transformation on $W^{\left(k\right)}=(w_{ij}^{\left(k\right)})$, and select the gene pairs with a z-score larger than 3, which are considered as the edges in the specific network $N^{\left(k\right)}$. That is to say, the nodes in the specific network $N^{\left(k\right)}$ are all the mutated genes. There is an edge between two genes if this gene pair has a z-score larger than 3, which means that this gene pair has a significantly specific weight in cancer k. Then, we obtain an unweighted specific network $N^{\left(k\right)}$ for detecting cancer specific driver modules.

#### 4.2.2. Cancer Specific Driver Module Detection

We first define the internal coverage, external coverage, and specific coverage of the specific driver module in cancer k. The large specific coverage denotes that the driver module has high internal coverage in a certain cancer and a high percentage of samples in this cancer, compared to other cancer types. Given a driver module $D=\{g_{1},g_{2},\cdots ,g_{I}\}$, the internal coverage of D in cancer k is defined as Equation (8),

(8) $$c\_in_{D}^{\left(k\right)}=\frac{\left|\underset{1\leq i\leq I}{\cup }\Gamma^{\left(k\right)}(g_{i})\right|}{m_{k}}.$$

The external coverage of D in cancer k is defined as Equation (9),

(9) $$c\_ex_{D}^{\left(k\right)}=\frac{\left|\underset{1\leq i\leq I}{\cup }\Gamma^{\left(k\right)}(g_{i})\right|}{\left|\underset{1\leq i\leq I}{\cup }\Gamma (g_{i})\right|},$$

and the specific coverage of D in cancer k is defined as Equation (10),

(10) $$c_{D}^{\left(k\right)}=\sqrt{c\_in_{D}^{\left(k\right)}\times c\_ex_{D}^{\left(k\right)}}.$$

We use the greedy algorithm to detect the specific driver modules from the specific network $N^{\left(k\right)}$ of cancer k. This greedy method protects the gene modules that have large external coverage in cancer k. The details of implementation of our greedy algorithm for detecting the specific driver module D of each gene g∈G in cancer *k* are shown as Algorithm 1.

We apply this greedy algorithm on each gene in specific network $N^{\left(k\right)}$ and consider the modules that have at least three genes as the specific driver modules. This greedy algorithm protects the gene pairs with high external coverage in cancer k, which guarantees high specific coverage of driver modules in a certain cancer to other cancer types.

**Algorithm 1** Cancer specific driver modules detection **Input**: $A^{\left(k\right)}$: mutation matrix of the kth caner type; A: mutation matrix for all cancer types; $N^{\left(k\right)}$: specific network for cancer k; g∈G: a gene in network $N^{\left(k\right)}$; **Output**: D: specific driver module of gene g. **Step 1:** Initialize D={g}. **Step 2:** Compute objective function f. (a) Compute the $f=c\_ex_{D}^{\left(k\right)}$ according to Equation (9). f is the objective function. **Step 3:** Update D and f. (a) Compute the $Neighbours(D)=\{g_{1},g_{2},\cdots ,g_{I}\}$. Neighbours(D) is a set of neighbours of D in $N^{\left(k\right)}$, and I is the number of neighbours.  (b) Compute the $\left\{c\_ex_{D\cup \{g_{1}\}}^{\left(k\right)},c\_ex_{D\cup \{g_{2}\}}^{\left(k\right)},\cdots ,c\_ex_{D\cup \{g_{I}\}}^{\left(k\right)}\right\}$ according to Equation (9). $c\_ex_{D\cup \{g_{i}\}}^{\left(k\right)}$ is the external coverage of *D* and its neighbour *g_i_*. (c) Select the gene $g_{i}\in Neighbours(D)$ with $c\_ex_{D\cup \{g_{i}\}}^{\left(k\right)}=\max_{1\leq j\leq I}\{c\_ex_{D\cup \{g_{j}\}}^{\left(k\right)}\}$ and $c\_ex_{D\cup \{g_{i}\}}^{\left(k\right)}>c\_ex_{D}^{\left(k\right)}$. (d) If $g_{i}$ exists, update $D=D\cup g_{i}$ and $f=c\_ex_{D\cup \{g_{i}\}}^{\left(k\right)}$. Then go to Step 2. If $g_{i}$ does not exist, return D.

#### 4.2.3. Evaluation Measures

***Specific coverage.*** We use the specific coverage to evaluate the specificity of specific driver modules, which is computed according to Equation (10). The large specific coverage denotes that the driver module has high internal coverage in a certain cancer and a high percentage of samples in this cancer compared to other cancer types. 

***Significance of mutual exclusivity.*** We also use the permutation test to assess the significance of mutual exclusivity in a certain cancer type. We permute the mutations of each gene among samples in a certain cancer type independently to hold the mutation frequency of each gene. Given a driver module $D=\{g_{1},g_{2},\cdots ,g_{I}\}$ for cancer k detected by CSDM, we calculate the real number of samples in which D is mutated. Then, we permute samples with mutations for each gene in cancer k independently for 1000 times. Then, the significance of mutual exclusivity, an empirical *p*-value, is the fraction of random samples with mutations in D larger than the real number of samples with mutations. The smaller the *p*-value is, the better. In this work, we consider the driver modules with the significance of mutual exclusivity *p*-value < 0.05 is significant. This *p*-value has 95% bootstrap confidence intervals. All *p*-values are corrected for multiple testing using the Benjamin–Hochberg method [71].

***Pathway Enrichment.*** We use the Gene Set Enrichment Analysis (GSEA) [48] to obtain the significance of a driver module based on the well-known pathway, which uses hypergeometric calculation to measure overlapping genes over all genes in the gene universe by the following formula,

(11) $$p-\mathrm{value}=\frac{\left(\begin{array}{l} K\\ k \end{array}\right)\left(\begin{array}{l} N-K\\ n-k \end{array}\right)}{\left(\begin{array}{l} N\\ n \end{array}\right)},$$

where N is the total number of genes, K is the number of genes in a well-known pathway, n is the number of genes in a driver module, and k is the number of overlapped genes both in the well-known pathway and the driver module. In our analysis, the reference gene set N is 45,956, which is used by GSEA [48]. After pathway enrichment, we consider the driver modules with statistical significance *p*-value < 0.05 as the true positive elements. All *p*-values are corrected for multiple testing using the Benjamin–Hochberg method [71].

***F-measure.*** For evaluating the performance of each method, we use F-measure to measure the accuracy of the driver modules based on well-known pathways, which are the gene sets of GO terms and KEGG pathways in this work. F-measure is the harmonic mean of precision and recall. The higher the F-measure, the more the driver modules can be enriched to the known pathways. The formulas of precision, recall, and F-measure are shown in the following:

(12) $$Precision=\frac{true\;positive}{true\;positive+false\;positive}$$

(13) $$Recall=\frac{true\;positive}{true\;positive+false\;negative}$$

(14) $$F-measure=2\times \frac{Precision\times Recall}{Precision+Recall}$$

## Figures and Tables

**Table 1:** Enriched pathways for BRCA driver module (*GATA3*, *ZNF703*, *CDH1*, *MAP3K1*, *FH*, *MAP2K4*, *LYZ*, *GFOD1*, and *CDKN1B*) performed by MITHrIL using standard KEGG pathways. The pathways are sorted by *p*-value, and only the pathways with *p*-value < 0.01 are selected.

Pathway	Impact Factor	*p*-Value
Pathways in cancer	11.282	2.134 × 10^−5^
Epithelial cell signaling in *Helicobacter pylori* infection	4.468	8.044 × 10^−4^
Hepatitis B	13.121	8.987 × 10^−4^
Epstein–Barr virus infection	7.806	1.101 × 10^−3^
HTLV-I infection	6.945	1.259 × 10^−3^
GnRH signaling pathway	9.044	2.054 × 10^−3^
ErbB signaling pathway	9.222	2.631 × 10^−3^
Small cell lung cancer	4.925	2.803 × 10^−3^
Neurotrophin signaling pathway	4.139	7.703 × 10^−3^

**Table 2:** Enriched pathways for BLCA driver module (*RXRA*, *ELF3*, *CDKN1A*, and *RHOA*) performed by MITHrIL using standard KEGG pathways. The pathways are sorted by *p*-value, and only the pathways with *p*-value < 0.01 are selected.

Pathway	Impact Factor	*p*-Value
Pathways in cancer	16.834	4.09 × 10^−8^
Melanoma	14.011	5.52 × 10^−4^
Glioma	14.099	6.58 × 10^−4^
Chronic myeloid leukemia	13.983	6.62 × 10^−4^
PI3K–Akt signaling pathway	11.242	8.61 × 10^−4^
HTLV-I infection	12.696	1.36 × 10^−3^
Proteoglycans in cancer	10.732	3.48 × 10^−3^
Hepatitis B	10.318	4.63 × 10^−3^
p53 signaling pathway	11.093	7.50 × 10^−3^
PPAR signaling pathway	5.895	9.08 × 10^−3^

## Supplementary Materials

The following are available online. Table S1: Percentages of overlaps between specific driver modules of different cancer types. Table S2. Percentages of overlapped genes between different cancer types.
