# Supplementary material for: Identifying Cancer Specific Driver Modules Using a Network-Based Method
Source: Molecules. 2018 May 8;23(5):1114. doi: 10.3390/molecules23051114 (PMC6100049; doi:10.3390/molecules23051114)
Supplement: Supplementary file 1 [file molecules-23-01114-s001.pdf]

Table S1. Percentages of overlapped modules between specific driver modules of different cancer types.

Cancer Type	BLCA	BRCA	COAD READ	GBM	HNSC	KIRC	LAML	LUAD	LUSC	OV	UCEC
BLCA											
BRCA	1.18E-04										
COADREAD	1.45E-04	1.95E-04									
GBM	1.32E-04	3.46E-04	2.25E-04								
HNSC	8.58E-04	4.57E-04	2.00E-04	3.76E-03							
KIRC	1.01E-04	1.07E-04	2.13E-04	3.76E-04	1.87E-04						
LAML	0	3.95E-03	1.81E-03	0	5.22E-05	8.15E-05					
LUAD	1.30E-04	3.20E-04	5.27E-04	3.85E-04	4.58E-04	3.85E-04	0				
LUSC	1.27E-04	2.06E-04	6.60E-05	2.07E-04	4.50E-04	6.48E-05	0	4.21E-03			
OV	8.66E-05	4.26E-03	3.09E-05	9.41E-05	9.64E-05	5.02E-05	0	1.04E-04	6.09E-05		
UCEC	1.39E-04	4.56E-04	1.41E-04	3.55E-02	3.52E-04	2.15E-04	6.39E-05	2.41E-04	1.63E-04	1.47E-04	

Table S2. Percentages of overlapped genes between different cancer types.

Cancer Type	BLCA	BRCA	COAD READ	GBM	HNSC	KIRC	LAML	LUAD	LUSC	OV	UCEC
BLCA											
BRCA	0.163										
COADREAD	0.102	0.119									
GBM	0.084	0.102	0.093								
HNSC	0.171	0.331	0.125	0.127							
KIRC	0.149	0.224	0.135	0.122	0.275						
LAML	0.000	0.000	0.001	0.000	0.000	0.000					
LUAD	0.055	0.103	0.035	0.038	0.098	0.045	0				
LUSC	0.072	0.168	0.058	0.076	0.186	0.096	0	0.096			
OV	0.003	0.005	0.001	0.002	0.003	0.002	0	0.003	0.002		
UCEC	0.159	0.288	0.115	0.115	0.344	0.266	0	0.071	0.154	0.004	
